# Editorial: COVID-19: the neurorehabilitation perspective

**DOI:** 10.3389/fneur.2023.1189295

**Published:** 2023-04-28

**Authors:** Thomas Platz, Nam-Jong Paik, David Good, Giorgio Sandrini

**Affiliations:** ^1^BDH-Klinik Greifswald, Institute for Neurorehabilitation and Evidence-Based Practice, An-Institut, University of Greifswald, Greifswald, Germany; ^2^Neurorehabilitation Research Group, University Medical Centre, Greifswald, Germany; ^3^Special Interest Group Clinical Pathways, World Federation for Neurorehabilitation, North Shields, United Kingdom; ^4^Seoul National University College of Medicine, Seoul National University Bundang Hospital, Seongnam, Republic of Korea; ^5^Penn State Milton S. Hershey Medical Center, Hershey, PA, United States; ^6^Department of Brain and Behavioral Sciences, University of Pavia, Pavia, Italy

**Keywords:** COVID-19, rehabilitation, telemedicine, health-care, pandemic, Low- and Middle-Income Countries (LMICs)

## COVID-19 and neuro-disabilities—A 2-fold consideration

On 11th March 2020, after a sharp increase in confirmed cases, the World Health Organization (WHO) announced the COVID-19 outbreak as a pandemic. Given the global public health threat, there was an urgent need for research and knowledge distribution on how best to manage patient care for those affected by the Coronavirus Disease 2019 (COVID-19).

Most people with COVID-19 have mild symptoms and recover, while 6.1% become critically ill (respiratory failure, septic shock, and/or multiple organ dysfunction/failure) (1) and might develop a “post-intensive care syndrome” (PICS) with motor, cognitive, and emotional disorders, necessitating intensive rehabilitation (2, 3). Indeed, multi-national observational studies indicated that neurological signs and symptoms can be observed in the majority of COVID-19 cases with a need for hospitalization (4). Equally important, even subjects with an initially mild course of COVID-19 reported symptoms that interfered with everyday life considerably over an extended period (5), i.e., for more than 4 weeks post-COVID-19 onset (called “Long COVID-19”) or more than 12 weeks (called “post-COVID-19”), again frequently including neurological symptoms (6). More recently, the Global Burden of Disease Long COVID-19 Collaborators (7) analyzed data from a total of 1.2 million individuals who had symptomatic SARS-CoV-2 infection. In the modeled estimates, 6.2% [95% uncertainty interval (UI), 2.4–13.3%] of these individuals experienced at least one of the three Long COVID symptom clusters, 3.2% (95% UI, 0.6–10.0%) reported persistent fatigue with bodily pain or mood swings, 3.7% (95% UI, 0.9–9.6%) reported ongoing respiratory problems, and 2.2% (95% UI, 0.3–7.6%) reported cognitive problems.

Hence, priority research was and is necessary to elaborate rehabilitative needs, therapeutic options, and managed care for those affected by COVID-19 and developing neurological impairments, i.e., “Neuro-COVID.”

Along these lines, research has to document the epidemiology and rehabilitation needs of COVID-19 cases and their clinical course. It should further address the effectiveness of neurorehabilitation treatment including the use of new technologies for home care purposes (e.g., the use of low-cost technologies such as smartphones or tablets for virtual medical examination, counseling, and tele-rehabilitation), as well as healthcare system questions (e.g., how to cope with rapidly increasing demands for services), and guidance (practice recommendations).

Furthermore, the effects of the mandated COVID-19 restrictions (social distancing) on people with neuro-disabilities (not caused by COVID-19) and their possibility to receive neurorehabilitation treatment have been major healthcare concerns in the field. Here again, research to elucidate such effects and to suggest means to overcome the detrimental effects of the mandated COVID-19 restrictions are of utmost importance since people with neuro-disabilities frequently have a long-term or ongoing need for therapy to support their physical, mental, and emotional wellbeing.

Accordingly, COVID-19-related practice recommendations have a 2-fold focus, one being rehabilitation to combat COVID-19 sequelae, and the second being rehabilitation during a pandemic and its restrictions imposed on those in need of rehabilitation (not caused by COVID-19) (8, 9).

To promote the rapid access to and exchange of COVID-19-related research relevant to neurorehabilitation, the World Federation for Neurorehabilitation (WFNR; wfnr.co.uk) initiated this Research Topic (RT) in collaboration with Frontiers. Overall, the RT attracted 25 articles with a broad scope of contents.

## Neurorehabilitation services—The impact of the COVID-19 pandemic, steps to be taken, and chances for the future

Recognizing the need to reorganize hospital and outpatient rehabilitation activities, the document by Bartolo et al. describes the measures adopted by the rehabilitation structures that first faced the fight against COVID-19 to inform all those who consequently found themselves involved in this rampant battle.

New forms to organize neurorehabilitation with the use of technology such as a “digital and artificial intelligence platform” (DAIP) were introduced and used effectively to cope with new affordances for rehabilitation services during the COVID-19 pandemic, providing qualitative support for goal-setting for remote consultations and reducing the time for scheduling and registering sessions (Saverino et al.).

A telemedicine process flow representing a replacement for in-person treatment and thereby the provision of equitable access to the care of vulnerable people was proposed by Matamala-Gomez et al. It has been conceptualized as a comprehensive service including (1) tele-assistance with patient counseling and medical treatment, (2) tele-monitoring of patients' health conditions and any changes over time, as well as (3) tele-rehabilitation, i.e., interventions to assess and promote body functions, activities, and participation, consecutively.

Survey data on professionals, adult patients, and children's caregivers' perceptions and satisfaction with tele-rehabilitation during the COVID-19 lockdown were presented by Assenza et al. indicating that tele-rehabilitation can indeed be a useful practice.

While such endeavors might serve to foster opportunities for improved healthcare (beyond the COVID-19 pandemic), nevertheless regulations against the spread of coronavirus (COVID-19) have not infrequently interrupted non-essential rehabilitation services globally.

As shown by the survey conducted by Surya et al. neurorehabilitation services were severely affected across India during the COVID-19 pandemic. Tele-neurorehabilitation has emerged as a new service delivery model during the pandemic and online means of education as the primary source of continuing medical education during the pandemic. Indeed, the pandemic situation has been seen to provide an opportunity to optimize the technological innovations in health and scale up these innovations to meet the growing burden of neurological disability in Low- and Middle-Income Countries (LMICs; Srivastava et al.).

## COVID-19 pandemic, specific clinical syndromes, and neurorehabilitation

Furthermore, in this Research Topic, specific recommendations were provided for specific clinical syndromes.

### Neuro-COVID-19

With severe COVID-19 infection, complex and long-lasting physical, cognitive, and functional impairments have often been observed after COVID-19. As outlined by Pincherle et al. early—defined as during and immediately after intensive care unit (ICU discharge)—rehabilitative interventions are fundamental for reducing the neurological burden of a disease that already heavily affects lung function as a possible long-term consequence.

Liguori et al. analyze the critical issues of COVID-19 on neuromuscular disease (NMD) and propose a home-based rehabilitation program targeted for this population after mild to moderate SARS-CoV-2 infection.

Sozzi et al. appropriately point out the need for neuropsychologists' intervention in taking care of COVID-19 patients, considering that this pandemic, in its manifestation as Neuro-COVID, may result in cognitive and behavioral alterations. Accordingly, all structures in which COVID-19 patients are hospitalized should be provided with information on cognitive, affective, and behavioral alterations resulting from this pathology, and integrate such knowledge into their patient care. Indeed, the findings of an observational study with COVID-19 and Post COVID-19 subjects with a need for rehabilitation highlight the gravity of neuropsychological and psychological symptoms that can be induced by COVID-19 infection and the need for tailored rehabilitation, including cognitive training and psychological support (Pistarini et al.). The paper by Mantovani et al. offers a perspective on the role of tele- and virtual rehabilitation to achieve adequate cognitive stimulation in the era of social distancing related to the COVID-19 pandemic.

### Pandemic-related restrictions and neurorehabilitation in general

With spasticity, prolonged suspension can potentially accelerate the morphological alterations connected (e.g., myotendinous and joint contractures and pain) which could potentially cause a long-term negative impact on the patient's level of activity and participation, as well as a deterioration in their quality of life. Several factors must be taken into account to guarantee both patients' necessary care and indications for minimizing the further spread of the pandemic. For this purpose, an *ad-hoc* treatment protocol was summarized by Baricich et al..

For the UK, it was demonstrated that both referrals to speech and language (SLT) services and access to SLT by patients were substantially less during the acute COVID-19 period in the UK than in the same period in 2019 (Chadd et al.). In addition, several service changes were common, including adopting more flexible approaches to provision (such as tele-therapy) and being unable to provide services to some patients.

Individuals with physical disabilities such as children with cerebral palsy could no longer benefit from physical rehabilitation during the pandemic. Using either a synchronous or asynchronous format, in collaboration with a therapist via tele-rehabilitation, Demers et al. suggest that active video games and low-cost virtual reality are promising delivery modes for at-home rehabilitation in the context of a global pandemic.

Another concern of pandemic-related restrictions is that people with neuro-disabilities have fewer opportunities for physical activities and training (aside from healthcare), being an essential part of their continuous efforts for wellbeing. The survey by Nightingale et al. investigated associations between physical activity and health-related quality of life outcomes in individuals with a neurological condition during government-mandated COVID-19 restrictions. The authors documented increased depression and fatigue, and a decrease in vitality with less leisure-time physical activity, highlighting the importance of and need to safely promote leisure-time physical activity during the COVID-19 pandemic in this at-risk population to help support health-related quality of life.

## Conclusions

The articles on the Research Topic “COVID-19—The Neurorehabilitation Perspective” collectively provide insights into the effects of COVID-19 on the nervous system, and hence, neurological manifestations called Neuro-COVID and rehabilitation needs to be addressed. In addition, the research demonstrates how—on a global level—mandatory restrictions during the pandemic affected rehabilitation services and, hence, the many people with neuro-disabilities in need of prolonged or ongoing treatment or other activities to promote and maintain their physical, mental, and emotional wellbeing. Finally, suggestions on how to promote telemedicine in neurorehabilitation, solutions being used, and user satisfaction all indicate technological options that might serve to generate a more widespread benefit of services well beyond the pandemic.

## Author contributions

TP designed and wrote the manuscript. All authors critically reviewed the manuscript for intellectual content. All authors contributed to the article and approved the submitted version.
